# Editorial: Mechanisms and strategies of unconventional antibody diversification for greater immune adaptability

**DOI:** 10.3389/fimmu.2023.1267556

**Published:** 2023-08-31

**Authors:** Jordan D. Dimitrov, Waithaka Mwangi, Xiaotian Zhong

**Affiliations:** ^1^ Centre de Recherche des Cordeliers, INSERM, CNRS, Sorbonne Université, Université Paris Cité, Paris, France; ^2^ Department of Diagnostic Medicine/Pathobiology, College of Veterinary Medicine, Kansas State University, Manhattan, KS, United States; ^3^ BioMedicine Design, Medicinal Sciences, Pfizer Worldwide Research & Development (R&D), Cambridge, MA, United States

**Keywords:** antibodies, immune diversification, post-translational modifications, therapeutic antibodies, immune adaptability, surrogate light chain, IVIG

Antibody repertoires are characterized by a vast diversity that endows antibodies with potential to recognize multifarious target antigens with high affinities and specificities. The diversities of antigen-binding sites of antibodies in human and mouse are generates by two principal processes –stochastic recombination of gene segments encoding variable regions of heavy and light immunoglobulin chains i.e., V(D)J recombination and random replacement of amino acid residues in variable regions, a process referred to as somatic hypermutation. Thesе diversification mechanisms are based on evolutionary principles and operate during different stages of B cell development in different tissue compartments. Thus, the V(D)J recombination occurs in bone morrow during early stages of B cell development, whereas the somatic hypermutation occurs in secondary immune organs and usually involves stimulation of B cells of antigen-specific T helper cells. These two processes can generate an enormous heterogeneity of antigen-binding sites (1). Since V(D)J recombination and somatic hypermutation processes operate in all adaptive immune responses, they can be considered conventional strategies for diversifying the antigen-binding specificities of antibodies. In most cases, they provide sufficient diversity of antigen-binding specificities of antibodies to successfully cope with pathogens. However, the theoretical diversity of antigenic molecules (antigenic space) in nature is limitless, and the sole implication of conventional strategies for diversification of antibody specificity may not be sufficient to provide full coverage of this diversity.

Not surprisingly, in addition to the conventional strategies for diversification of antibody specificities immune system of humans can employ a set of other mechanisms contributing to the variability of antigen binding sites. These strategies are usually employed only in particular conditions and by a fraction of antibodies in the repertoires. Consequently, they have been referred to us as unconventional diversification strategies (2). The unconventional strategies can be categorized in different groups (2). They include, the integration of non-immunoglobulin proteins or part of proteins in variable regions (3–5), post-translational modifications in variable regions, such as sulfation of tyrosine (6–8), cysteinylation (9) and glycosylation (10), the use of metal ions or heterocyclic cofactor molecules as interfacial cofactors (11–13), as well as noncanonical structural reconfigurations of the antigen binding sites of antibodies (14, 15). A common feature of these strategies for diversification of antibody specificities is that they modify the structural configuration of the antigen-binding site. Thus, in the case of post-translational modifications or the use of cofactor molecules, antibodies can simultaneously utilize the chemical and steric diversity intrinsic to amino acid residues composing the polypeptide chain, in addition to the unique chemistries and topologies provided by sulfate groups, glycans, or metal ions. This reshaping of the antigen binding site can permit binding to antigens that are otherwise difficult to be targeted by the conventional mechanisms. It is noteworthy that antibodies that utilize these unconventional mechanisms for diversification of immune specificity are often detected following chronic immune stimulation or in the case of highly genetically variable pathogens such as HIV-1 (2). This observation suggests that immune system employs the unconventional mechanisms as an adaptation strategy in situations where canonical configurations of antigen binding sites might not be suitable for efficient pathogen neutralization.

The articles published in the journal Research Topic “*Mechanisms and strategies of unconventional antibody diversification for greater immune adaptability*” shed further light on mechanistic and functional aspects of unconventional mechanisms of diversification of antibody specificities. In their study, van de Bovenkamp et al. demonstrated with series of elegant experiments that N-linked glycans on Fab have an impact on the thermodynamic stability of human antibodies. This study unveiled a novel function of Fab-linked glycans. Indeed, previous studies (10, 16) demonstrated that Fab glycans can contribute directly to the antigen binding specificity of antibodies through bulk size space filling or charge-charge interaction. One study also demonstrated that the glycan plays a role for blocking of targeted antigens by steric interference (17). Importantly, van de Bovenkamp et al.‘s study used therapeutic antibodies as a model to assess the role of glycans on stability. The results suggest that the addition of a glycosylation site in the anti-TNF alpha antibody Adalimumab can enhance the thermodynamic stability of the antibody. The authors hypothesized that glycans can increase antibody stability by shielding hydrophobic patches. Since stability is an important characteristic for the development of therapeutic antibodies, this data suggests an exploration of an engineering strategy for antibodies.

In their article, Zhong and D’Antona provide a comprehensive analysis of the role of tyrosine sulfation in CDR regions for regulating the potency of antigen binding, as well as for diversifying immune specificities. The authors present well-selected examples from studies of different antibodies that utilize tyrosine sulfation for the neutralization of HIV-1. Notably, the review analyzes the potential use of this post-translational mechanism to diversify immune specificity for the rational development of therapeutic antibodies. The technical and intellectual challenges of this goal are discussed. Considering the current limitations of tyrosine-sulfation detection methodologies, along with the frequent presence of tyrosine residues in antibody CDRs, the authors propose that the tyrosine-sulfation modification is significantly under-detected in therapeutic antibodies.

In their review article, von Gunten et al. present an overview of the roles of both conventional and unconventional diversification mechanisms in the therapeutic mechanisms of pooled human immunoglobulins. The authors discuss the systemic repercussions of various diversification mechanisms, including post-translational modifications of immunoglobulins, on the therapeutic activity and function of IgG and IgA. This discussion suggests that unconventional mechanisms offer an opportunity for optimizing and tailoring the immune-regulatory functions of pooled therapeutic immunoglobulins.

In their article, Ott et al. performed meticulous analyses of the evolution of light surrogate chains in different species. This article also highlighted the functional roles of different components of the light chain, VpreB1 and IGLL1. Notably, the work presented comprehensive details about how the surrogate light chain exerts quality control over the diversity of the heavy chain during the early stages of B cell development, underlining the important role of this component in the evolution of the diversity of immune specificity in later stages of B cell development. Moreover, the study by Ott et al. demonstrated a positive relationship between the size of the CDR H3 region and the length of VpreB1.

In conclusion, the Research Topic presents valuable articles that expand our knowledge about the unconventional mechanisms of diversification of immune specificities. They reflect numerous recent technical and intellectual advancements in the studies of human antibody repertoires and provide the basis for further research in this direction. These articles also uncover the potential for the rational use of unconventional strategies for the development of novel or improved antibody-based therapeutics.

## Author contributions

JD: Conceptualization, Writing – original draft, Writing – review & editing. WM: Conceptualization, Writing – original draft, Writing – review & editing. XZ: Conceptualization, Writing – original draft, Writing – review & editing.
